# Correction: Mahnashi et al. In-Vitro, In-Vivo, Molecular Docking and ADMET Studies of 2-Substituted 3,7-Dihydroxy-4H-chromen-4-one for Oxidative Stress, Inflammation and Alzheimer’s Disease. *Metabolites* 2022, *12*, 1055

**DOI:** 10.3390/metabo15080532

**Published:** 2025-08-06

**Authors:** Mater H. Mahnashi, Mohammed Abdulrahman Alshahrani, Mohammed H. Nahari, Syed Shams ul Hassan, Muhammad Saeed Jan, Muhammad Ayaz, Farhat Ullah, Osama M. Alshehri, Mohammad Ali Alshehri, Umer Rashid, Abdul Sadiq

**Affiliations:** 1 Department of Pharmaceutical Chemistry, College of Pharmacy, Najran University, Najran 61441, Saudi Arabia; mhmahneshi@nu.edu.sa; 2 Department of Clinical Laboratory Sciences, Faculty of Applied Medical Sciences, Najran University, Najran 61441, Saudi Arabia; mhnahari@nu.edu.sa (M.H.N.); usamah2012@gmail.com (O.M.A.); 3 Shanghai Key Laboratory for Molecular Engineering of Chiral Drugs, School of Pharmacy, Shanghai Jiao Tong University, Shanghai 200240, China; shams1327@yahoo.com; 4 Department of Natural Product Chemistry, School of Pharmacy, Shanghai Jiao Tong University, Shanghai 200240, China; 5 Department of Pharmacy, Bacha Khan University, Charsadda 24420, KP, Pakistan; saeedjanpharmacist@gmail.com; 6 Department of Pharmacy, Faculty of Biological Sciences, University of Malakand, Dir (L), Chakdara 18000, KP, Pakistan; mayaz@uom.edu.pk (M.A.); drfarhatullah@uom.edu.pk (F.U.); 7 Medical Genetics Laboratory Sciences, Faculty of Applied Medical Sciences, Najran University, Najran 61441, Saudi Arabia; maalshehri@nu.edu.sa; 8 Department of Chemistry, COMSATS University Islamabad, Abbottabad Campus, Abbottabad 22060, KP, Pakistan

## Text Correction

There was an error in the original publication [1]. The authors request to delete Reference [83] and replace the [83] “Xu, S.; Tao, H.; Cao, W.; Cao, L.; Lin, Y.; Zhao, S.-M.; Xu, W.; Cao, J.; Zhao, J.-Y. Ketogenic diets inhibit mitochondrial biogenesis and induce cardiac fibrosis. *Signal Transduct. Target. Ther.* **2021**, 6, 54” with Reference [61] “Wang, D.; Zhao, R.; Qu, Y.-Y.; Mei, X.-Y.; Zhang, X.; Zhou, Q.; Li, Y.; Yang, S.-B.; Zuo, Z.-G.; Chen, Y.-M.; et al. Colonic Lysine Homocysteinylation Induced by High-Fat Diet Suppresses DNA Damage Repair. *Cell Rep.* **2018**, 25, 398–412”.

A correction has been made to Section 4, Paragraph 6:

Free radicals are generated during metabolic processes in the body and are subsequently neutralized by natural antioxidant system like catalases and hydro-peroxidases [79,80]. However, in case of excessive free radical production or the body’s inability to scavenge the free radicals effectively, supplementation of exogenous antioxidants is extremely necessary [81]. In AD, Aβ proteins are deposited in the brain, which is considered as a mitochondrial poison. After production, it readily attacks lipids, membranes, proteins and nerve cells, causing genetic mutations, cells deterioration and the disruption of energy production by mitochondria [82]. Subsequently, supplementation of antioxidants is an integral part of the drug combinations for AD patients. Natural products and their derived compounds have received considerable attention as potential multi-target agents. Of particular interest is polyphenols which, being part of natural products and nutraceuticals, can delay the aging process and can act on multiple targets of AD. Numerous flavonoids are reported to inhibit cholinesterases, BACE1, scavenge free radicals and act as anti-inflammatory agents [61]. In the current study, our test compound showed efficacy on multiple targets and thus acts as a multi-target lead compound. However, further detailed studies regarding the bioavailability and in-vivo efficacy are required.

The authors state that the scientific conclusions are unaffected. This correction was approved by the Academic Editor. The original publication has also been updated.
