# Supplementary material for: In-Vitro, In-Vivo, Molecular Docking and ADMET Studies of 2-Substituted 3,7-Dihydroxy-4H-chromen-4-one for Oxidative Stress, Inflammation and Alzheimer’s Disease
Source: Metabolites. 2022 Nov 2;12(11):1055. doi: 10.3390/metabo12111055 (PMC9694897; doi:10.3390/metabo12111055)

# In-vitro, In-vivo, Molecular Docking and ADMET Studies of 2-substituted 3,7-dihydroxy-4H-chromen-4-one for Neuroinflammation

**Figure S1:** $^{1}$H NMR spectrum of isolated 2-(3,4-dimethoxyphenyl)-3,7-dihydroxy-4H-chromen-4-one.

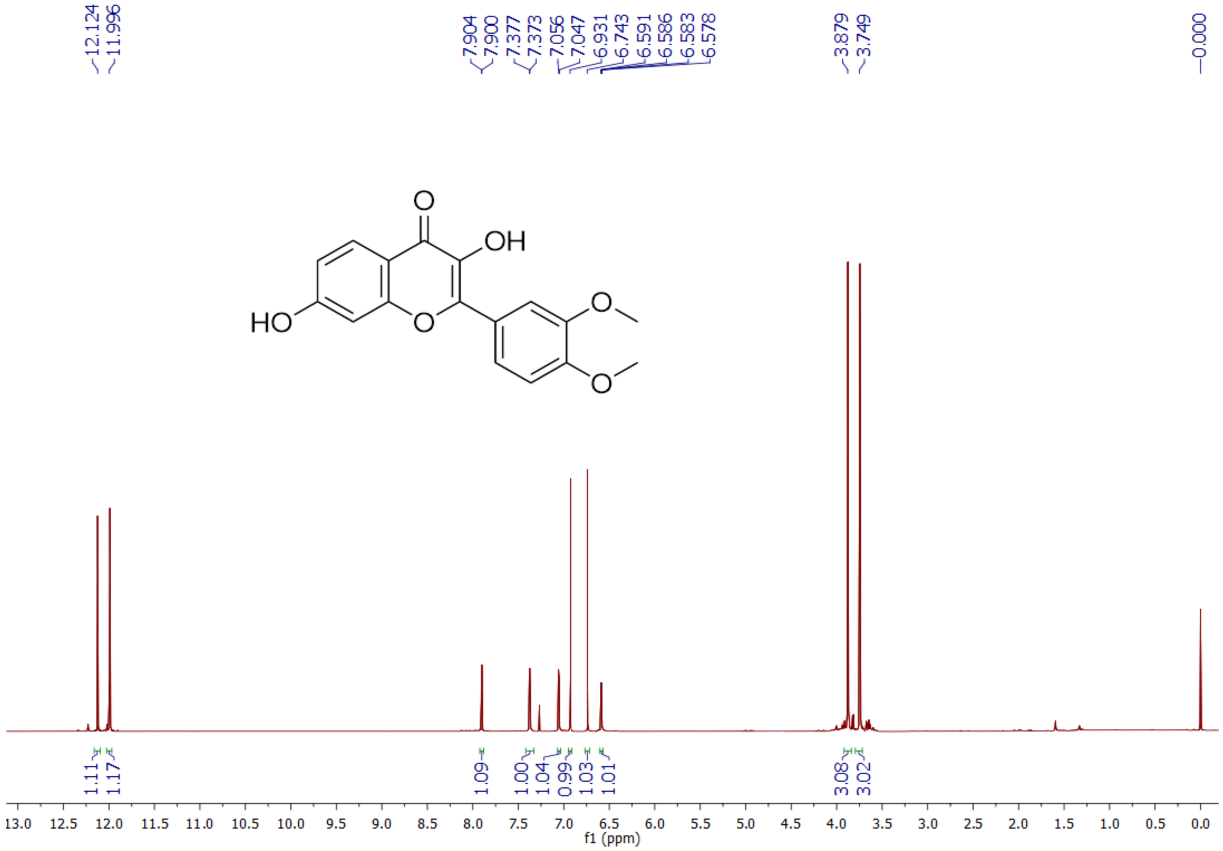

**Figure S2:** $^{13}C$ NMR spectrum of isolated 2-(3,4-dimethoxyphenyl)-3,7-dihydroxy-4H-chromen-4-one.

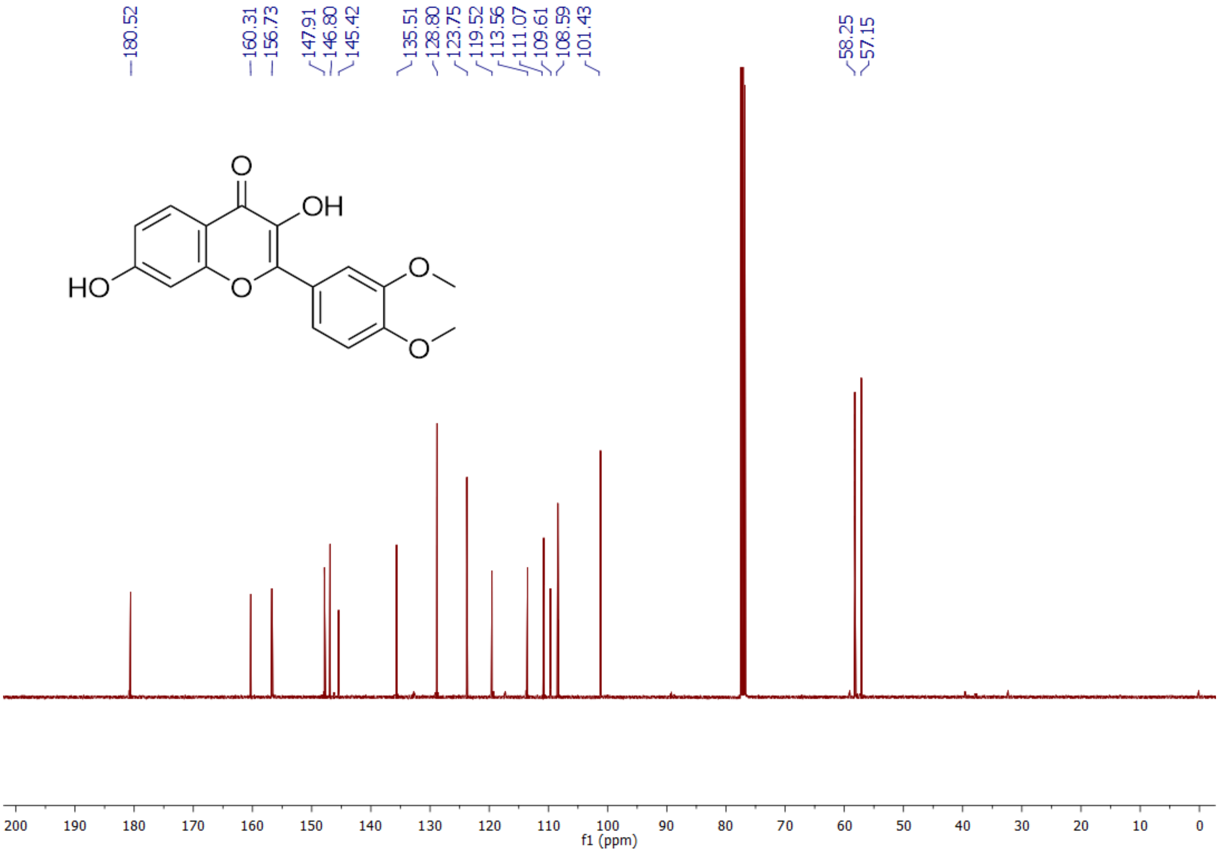


**Figure S3:** MS spectrum of isolated 2-(3,4-dimethoxyphenyl)-3,7-dihydroxy-4H-chromen-4-one.

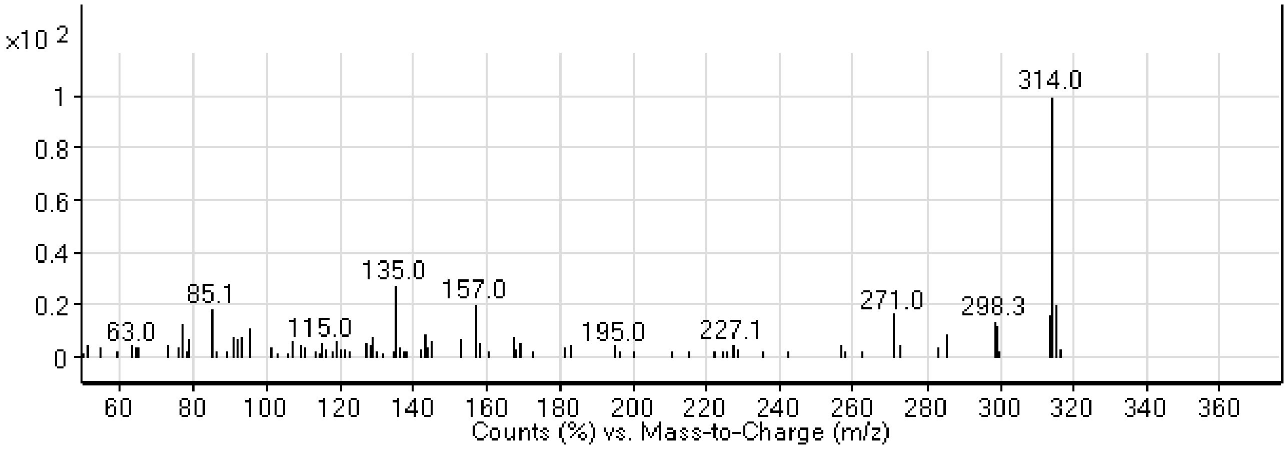

Supplement: Supplementary file 1 [file metabolites-12-01055-s001.zip › metabolites-1938840-supplementary.pdf]
